# The Commission of the French Academy on the Ferruginous Bread of M. Derouet-Boissière

**Published:** 1842-05-07

**Authors:** 


*On the Ferruginous Bread of M. Derouet-Boissière.* By the commission of the French Academy.—M. Derouet-Boissière having been extremely successful in curing obstinate cases of amenorrhœa and chlorosis, which had resisted all other means of treatment, by means of bread impregnated with minute doses of iron, a committee of the Academy of Medicine was appointed to report on the subject. They accordidgly saw bread prepared with small quantities of lactate, acetate, and protocarbonate of iron, and proceeded in the public hospitals to make trial of its powers. The bread was prepared in the proportion of 3½ ounces of bread to 3 or 4 grains of the salts of iron, and it is stated that when the iron did not exceed this quantity, the bread had no disagreeable flavour, though the chalybeate taste was slightly apparent.

During the months of November and December, 1840, seven chlorotic patients were treated with this bread in the Hotel Dieu. They were allowed two small loaves of this bread daily, each containing about three grains of the lactate of iron, and occasionally an equal quantity of sub-carbonate. Of the seven chlorotic females, five had the disease in a very advanced stage, the other two were in a state of anemia, brought on by repeated and copious evacuations of blood. These last two were not able to take the quantity of bread allowed them at first, but gradually became able to do so, and left the hospital cured, the one after six weeks of treatment, the other after two months. Their menstrual discharges were by that time completely re-established.

Two of the confirmed cases of chlorosis were sensibly improved after the use of the bread for six days, and one was dismissed cured in a fortnight, the other in a month.

The other three cases were old confirmed cases, which had resisted the usual treatment employed for the cure of this disease, and their cases are given in detail. In the first of these cases the treatment by the ferruginous bread was begun on the 20th of November, and by the 6th of December the menstrual secretion appeared and continued for three days. She was nevertheless kept in the hospital for another month, when, having become quite regular, and all symptoms of chlorosis having disappeared, she was dismissed. In the second case the ferruginous bread was begun to be administered on the 4th of December. On the 10th she was less pale and felt better. On the 24th the menses appeared and continued four days. She rapidly improved from this date, but was detained in the hospital till the next menstrual period was over, when she was dismissed cured. The third case was the most severe, and the symptoms of chlorosis of longest duration; the health seemed completely broken. The treatment commenced on the 20th of December, and she was allowed two of the small loaves daily. For the first fifteen days there was little apparent improvement, but from this date the symptoms of amendment became obvious. Her pallid cheeks gradually acquired a little colour, she improved in health and strength, and was dismissed with all the marks of health on the 22d of February.

Soon after this chalybeate bread was administered, the committee were able to detect the presence of iron in the urine, but its appearance there seemed to be inconstant, as they would often detect it at one time, and not at another.

The committee from these trials concluded, that this is an excellent mode of administering chalybeates; that, when in the small proportion of two or three grains of this salt to the loaf of three ounces and a half, its presence cannot be detected by any change of colour; that the salt of iron used is not decomposed by the process of baking; and that the iron can always be detected in it by the use of the ordinary reagents.

It may be remarked, that M. Derouet-Boissière was first led to administer iron along with the food, from the conviction that it would be more likely to be taken into the circulation, by being absorbed along with the chyle, than if given according to the usual methods.—*Ibid.*, from ***Bulletin de l'Academie Royale de Medecine.*** April, 1841.

